# Suicide in People With Co‐occurring Substance Use and Mental Health Conditions: An Integrative Literature Review to Inform Risk Formulation in Mental Health Nursing

**DOI:** 10.1111/inm.70375

**Published:** 2026-10-13

**Authors:** Rebecca Marshall, Anita Green, Magdalena Zasada, Caroline Nicholson

**Affiliations:** ^1^ Faculty of Health Sciences University of Surrey Guildford UK; ^2^ Sussex Partnership NHS Foundation Trust Worthing UK

**Keywords:** dual diagnosis, mental disorders, risk assessment, risk factors, suicide

## Abstract

People with co‐occurring substance use and mental health conditions experience disproportionately high levels of suicidal ideation, attempts and deaths compared with those with either condition alone, yet guidance for suicide risk formulation in this group is limited. This integrative literature review synthesised evidence on suicidality in adults with co‐occurring conditions, identified risk and protective factors, and refined risk formulation approaches for mental health nursing. Using Whittemore and Knafl's methodology and Toronto and Remmington's stepwise guidance, CINAHL, Embase, Medline and PsycInfo were searched without date limits. Empirical studies of adults with co‐occurring substance use and mental health conditions were included, alongside relevant grey literature; specialist populations (e.g., prison, veteran, neurological/developmental) were excluded. Thirty‐three quantitative studies and six grey literature sources (total *n* = 39; 35 883 participants across 19 countries) were appraised using Critical Appraisal Skills Programme tools and the AACODS checklist. Co‐occurring conditions were consistently associated with elevated suicidality, especially mood disorders, notably depression, combined with alcohol use disorder. Risk factors were mapped onto an extended 5Ps model: presenting, predisposing, precipitating, perpetuating and protective domains, with an additional Patient Experience domain to capture barriers to care, service dissatisfaction and stigma. Protective factors were comparatively scarce. Applying the Integrated Motivational–Volitional model highlighted psychological processes, including mental pain, perceived burdensomeness, impulsivity and access to means, that may contribute to suicidal behaviour. For mental health nurses and other mental health professionals, incorporating patient experience and IMV‐informed processes into an extended 5Ps framework may support more thorough suicide risk formulation and the development of safety and treatment plans that promote safer, more equitable and integrated care for people with co‐occurring conditions.

## 1 Introduction

Co‐occurring substance use and mental health conditions (previously termed ‘dual diagnosis’) are common in mental health services and are associated with elevated suicide risk. In England, nearly half of those who die by suicide have a history of problematic alcohol use and over one‐third have a history of problematic drug use (an unreported proportion will have been using both alcohol and drugs and will be accounted for in both percentages), with even higher proportions among mental health patients (National Confidential Inquiry into Suicide and Safety in Mental Health [NCISH] 2025). Despite this, service responses remain fragmented, and specific guidance for suicide risk assessment in this population is limited.

Co‐occurring conditions encompass a wide spectrum of mental health diagnoses and symptoms, combined with diverse patterns of alcohol and drug use, different levels of dependence and states of intoxication (Public Health England 2017). Studies examining the prevalence of co‐occurring conditions suggest that 30%–50% of people with serious mental illness also have a co‐occurring drug or alcohol problem (Regier et al. 1990; Weaver et al. 2001). The Comorbidity of Substance Misuse and Mental Illness Collaborative study found that 44% of mental health service users reported hazardous or harmful alcohol or drug use within the previous year (Weaver et al. 2003). Recent articles cite these studies when providing prevalence estimates, further demonstrating the lack of current research on co‐occurring conditions.

Globally, suicide is a leading cause of death, with an estimated 727 000 deaths in 2021 (World Health Organisation [WHO] 2025). In the United Kingdom, 26% of suicides between 2012 and 2022 occurred among people who had been in contact with mental health services in the preceding year (NCISH 2025). Alcohol and drug use are highly prevalent in this group, and the national suicide prevention strategy for England (Department of Health and Social Care [DHSC] 2023) identifies people in contact with mental health and substance use services as a key priority group.

Historically, people with co‐occurring conditions have often been required to address their substance use before being accepted for mental health treatment (Care Quality Commission 2015; Making Every Adult Matter 2022), despite evidence that substances often function as coping mechanisms in the context of unaddressed mental distress (National Institute for Health and Care Excellence [NICE] 2016). NCISH (2024) reported that 70% of people who died by suicide whilst under the care of alcohol and drug services had not been in contact with mental health services in the preceding year, prompting recommendations for joint working between drug and alcohol and mental health services and routine assessment of substance use within mental health settings.

Various models of risk assessment are used by organisations providing mental health care, and the forms used vary between services. NCISH (2018) examined the use of risk assessment tools across UK mental health services. All 85 providers of mental health care were asked to describe the tools they used, and clinicians, patients and carers were invited to complete an online survey. The study found little consistency in the length, content or use of risk tools. Most were checklists intended to predict future behaviour, with scores used to inform management decisions. However, risk scales have limited value in guiding risk management (Large et al. 2017; Oates 2018) and poor predictive validity (Chan et al. 2016; Quinlivan et al. 2014, 2016; Steeg et al. 2018). NCISH (2018) therefore recommended an approach that incorporates a formulation of risk based on the person's current situation, history and social circumstances. It also recommended training staff to undertake formulation collaboratively, personally and individually with the person and, where appropriate, their family or carer.

The 5Ps model of risk formulation is widely used in UK mental health services. Weerasekera (1993) initially proposed the model following a literature review that identified the need for a comprehensive and memorable approach to formulation. Macneil et al. (2012) subsequently developed the model, which considers presenting problems, predisposing, precipitating, perpetuating and protective factors (Table 1).

**TABLE 1:** 5Ps risk formulation framework (Macneil et al. 2012).

Risk categories	Description
Presenting problem	This is a detailed description of the difficulties the individual is currently experiencing. It includes their thoughts, feelings, and behaviours
Predisposing factors	Historical and background factors that can make someone more vulnerable to mental health issues, including genetics, early life experiences, or other long‐term vulnerabilities
Precipitating factors	Immediate triggers or events that seem to have led to the onset of the problem. Examples include a loss of a loved one, a major life change, or a significant stressor
Perpetuating factors	These are the factors that keep the problem going. They can be ongoing unhelpful coping strategies, substance use, or recurring patterns of thought or behaviour
Protective factors	These are the internal and external strengths and resources that help an individual cope and maintain their mental well‐being. They can be personal traits, supportive relationships, or activities like hobbies

In practice, substance use is frequently recorded in a single section, usually as a perpetuating factor that interferes with treatment. Given the heightened suicide risk in people with co‐occurring conditions, this conceptualisation and approach is likely to be overly simplistic and may lead to unsafe decisions regarding mental health care.

## 2 Research Question

The research question was developed to further understanding of the reasons for the elevated suicide risk in this patient group and to explore how risk can be better recognised, identified and responded to within mental health settings.

What risk factors for suicidality are associated with co‐occurring substance use and mental health conditions, and how can these inform clinical risk formulation in mental health services?

## 3 Aims

This review aimed to:

1. *Synthesise evidence* on factors associated with suicidality in adults with co‐occurring substance use and mental health conditions.
2. *Apply these findings* to a formulation framework widely used in mental health services (the 5Ps) and critically appraise its adequacy in this context.
3. *Propose an enhanced risk formulation model* that accounts for the complex historic and current factors related to increased suicide risk in this patient group across all risk domains, with a sixth domain (Patient Experience), informed by the Integrated Motivational–Volitional (IMV) Model of Suicidal Behaviour.
4. *Identify gaps in the literature* and highlight implications for mental health nursing practice and future research.

## 4 Methods

### 4.1 Design

An Integrative Literature Review (ILR) was chosen because both suicidality and co‐occurring conditions are conceptually broad and studied using heterogeneous designs. A conventional narrow systematic review (e.g., focusing on one diagnosis–substance pairing) would have overlooked the complexity encountered in clinical practice. The ILR approach, as outlined by Whittemore and Knafl (2005) and elaborated by Toronto and Remington (2020), allows inclusion of diverse methodologies and grey literature, supporting practice‐oriented synthesis.

The clinical problem was identified through practice experience in UK mental health services and repeated NCISH reports highlighting the high proportion of suicide deaths among those with problematic use of substances combined with a lack of specific guidance on suicide risk assessment for this group.

### 4.2 Search Strategy

Searches were conducted in Medline, CINAHL, PsycInfo and Embase using controlled vocabulary and keywords relating to dual diagnosis/co‐occurring conditions and suicide/suicidality. The primary MeSH term was ‘dual diagnosis (psychiatry)’ combined with terms such as ‘suicide’, ‘suicidal ideation’, ‘suicide attempts’ and ‘suicidology’. Alternative co‐occurring condition terms were piloted but yielded large numbers of irrelevant results; therefore, ‘dual diagnosis’ was retained as the most specific indexing term. Search terms for each database are presented in Table 2.

**TABLE 2:** Database search.

Database	Search terms—fields—all
MEDLINE Results = 136 Adult (19+) = 113	(MH “Diagnosis, Dual (Psychiatry)”) AND ((MH “Suicide”) OR (MH “Suicide, Completed”) OR (MH “Suicide, Attempted”))
CINAHL Results = 74 All adult = 48	(MH “Diagnosis, Dual (Psychiatry)”) AND (MH “Suicide, Attempted”) OR (MH “Suicide”) OR (MH “Suicide Prevention”) OR (MH “Suicide Risk (Saba CCC)”) OR (MH “Suicide Prevention (Iowa NIC)”) OR (MH “Suicide Self‐Restraint (Iowa NOC)”)
PsychInfo Results = 20 Adult (18+) = 13	(DE “Dual Diagnosis”) AND (DE “Suicidality” OR DE “Suicide” OR DE “Suicidology”)
Embase	Psychiatric dual diagnosis/OR mental disease/AND substance abuse/AND suicide attempt/or suicide/or suicide prevention/
Results = 1350	(full text and human and “remove medline records”)
Adult = 274	(‘psychiatric dual diagnosis’/exp. OR ‘psychiatric dual diagnosis’) AND (‘suicide attempt’/exp. OR ‘suicide attempt’) OR ‘suicide’/exp. OR ‘suicide’

No date limits were applied because no prior review was identified that specifically addressed suicidality in the context of co‐occurring conditions. A search of the Cochrane Library confirmed the absence of relevant systematic reviews.

Grey literature was not subject to the database search and was identified through discussion with Progress (Co‐Occurring Mental Health, Alcohol & Drugs), a UK‐based national consortium of senior clinicians in co‐occurring conditions, and through targeted searches of key organisations (NCISH, NICE, Royal College of Psychiatrists, DHSC, Public Health England).

### 4.3 Inclusion and Exclusion Criteria

Table 3 summarises the criteria used. Studies were included if they:

- Focused on adults with co‐occurring substance use and mental health conditions (diagnosed by ICD/DSM or equivalent), and
- Reported suicidality, defined as suicidal ideation, suicide attempts, or suicide deaths.

**TABLE 3:** Inclusion and exclusion criteria.

Domain	Inclusion	Exclusion
Population	Adults with co‐occurring substance use and mental health conditions and suicidality	Under 18 s; veterans; prison populations; specific neurological or developmental conditions
Topic	Co‐occurring substance use and mental health; suicidality (ideation, attempts, deaths)	No suicidality reported; no specific data for co‐occurring conditions
Setting	Community and inpatient mental health services, drug and alcohol services, general population samples	Prison settings
Study type	Quantitative, qualitative or mixed‐methods empirical studies; relevant grey literature	Genetic studies; machine‐translated articles; abstract‐only reports
Language	English	Non‐English or machine‐translated

Exclusions:

Studies including participants aged under 18 years were excluded. Studies of prison populations were also excluded because this review examines co‐occurring conditions and suicide in mental health care rather than custodial settings. Incarceration is associated with an increased suicide rate (Zhong et al. 2021), and prisons in the UK operate within a distinct care system tailored to the custodial environment.

Studies that solely focus on participants with acquired or traumatic brain injury were excluded because brain injury introduces distinct neurocognitive, behavioural, and physiological changes that substantially alter the mechanisms underlying suicidality. Individuals with brain injury have been shown to have a 2.23 times higher rate of suicide attempts (Lu et al. 2020).

Studies focusing solely on veterans were excluded because there are variable rates of suicide in this group dependent on nationality. For example, the suicide rate among US veterans is 1.5 times higher than the general population (Ruiz et al. 2022), whereas in the UK the rate is similar to those who have never served in the armed forces (Rodway et al. 2023). The studies found in the search related largely to US veterans.

Many included studies may nevertheless have contained individual participants with a history of incarceration, diagnosed or undiagnosed brain injury, developmental conditions or veteran status, unless the study explicitly excluded these groups. The exclusion criteria applied only to studies focused solely on those specialist populations.

- Studies where tobacco was the sole substance of use, where substances were prescribed as part of the study, or where results were not specific to people with co‐occurring conditions.
- Articles not reporting suicidality and those available only as abstracts or machine‐translated texts.

### 4.4 Screening and Selection

The database search yielded 448 records, of which 47 were duplicates. The titles and abstracts of the remaining 401 records were screened against the inclusion and exclusion criteria, yielding 85 potentially eligible studies. Full texts were retrieved for 82 studies. To validate the primary reviewer's decisions in accordance with Covidence (2025) guidance for systematic reviews, a second reviewer (C.J.N., M.Z. or A.G.) independently screened a 20% sample of the full‐text articles. Thirty‐three empirical studies met the inclusion criteria, including one identified through reference searching. Six grey‐literature sources were also included, resulting in a total of 39 publications. The selection process was documented in a PRISMA‐style flow diagram (Page et al. 2021; Haddaway et al. 2022; Figure 1).

**FIGURE 1:**
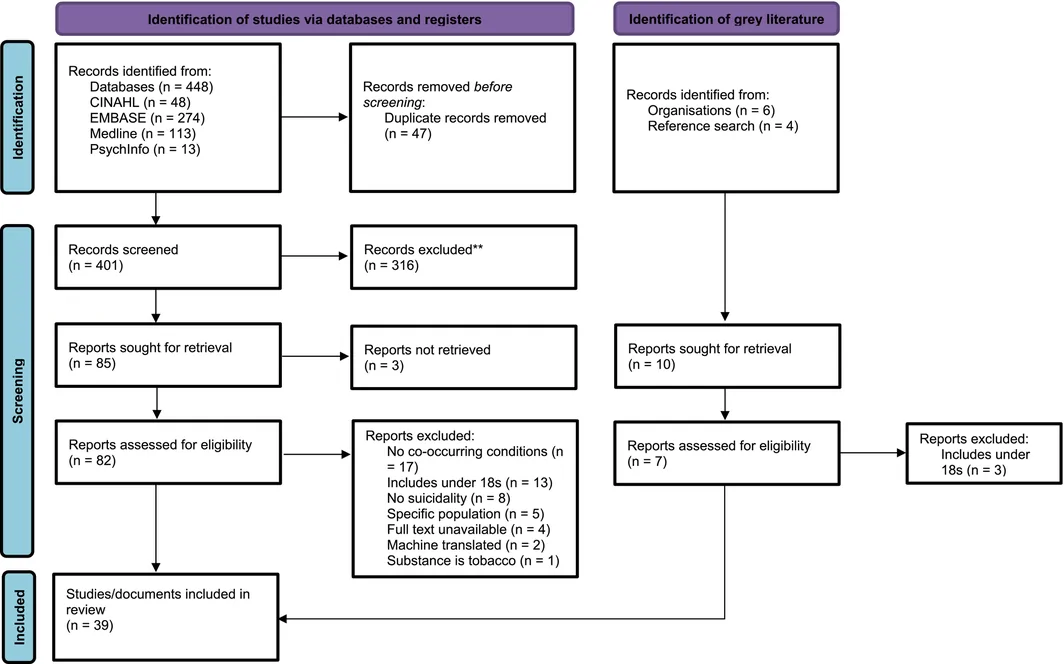
PRISMA flow diagram.

### 4.5 Quality Appraisal

All empirical studies used quantitative observational designs: 14 case–control, 14 cross‐sectional and five cohort studies. Appropriate CASP checklists were used for appraisal (Critical Appraisal Skills Programme 2023). The review forms part of a doctoral project and was led by the primary reviewer. Although duplicate appraisal of every study is considered best practice, the project's infrastructure and timescale did not permit this. Instead, 20% of included articles were independently double‐appraised by A.G., producing good inter‐rater reliability. This partial double‐appraisal strategy was specified in the review protocol registered with PROSPERO.

Each study was rated across domains of validity, results and relevance to the local population and was then assigned an overall quality rating of low, medium or high. Section ratings were determined as follows. A section was rated high quality when all answers to its checklist questions were ‘yes’. It was rated medium quality when half of the answers were ‘no’ or ‘can't tell’, and low quality when more than half were ‘no’ or ‘can't tell’. When most categories were rated at the same level, the overall study rating reflected that level. When categories were divided equally between two levels, the lower rating was assigned. Table 4 summarises the quality ratings for each study. Red, amber and green colours have been added to the table to indicate low, medium and high quality respectively to aid quick, visual representation.

**TABLE 4:** CASP checklist quality ratings.

Study	CASP checklist rating				
	Validity (Section A)	Results (Section B)	Utility to local population (Section C)	Methodological rigour	Overall quality rating
Agosti and Levin (2006)	Medium	High	High	Medium	Medium
Anestis et al. (2012)	Medium	Medium	Medium	Medium	Medium
Blixen et al. (1997)	Medium	High	High	Medium	Medium
Carballo et al. (2007)	Low	Low	Medium	Low	Low
Cornelius et al. (1995)	Medium	Medium	Medium	Medium	Medium
Davis et al. (2005)	High	High	High	High	High
De Bernardo et al. (2002)	Low	Low	Low	Low	Low
Driessen et al. (1998)	Medium	Medium	Medium	Medium	Medium
Dumais et al. (2005)	High	Medium	High	High	High
Erfan et al. (2010)	Medium	Medium	Medium	Medium	Medium
Fitzsimons et al. (2007)	High	Medium	Medium	Medium	Medium
Garlow et al. (2003)	Medium	High	High	Medium	Medium
Grant and Hasin (1999)	Medium	High	High	Medium	Medium
Hakko et al. (2005)	High	Medium	High	Medium	Medium
Handley et al. (2016)	High	High	High	High	High
Harned et al. (2006)	Medium	Medium	Low	Medium	Medium
Landheim et al. (2006)	Medium	High	High	Medium	Medium
Leposavić et al. (2015)	Medium	Low	Medium	Medium	Medium
Marín‐Navarrete et al. (2016)	Medium	High	Medium	High	Medium
Matsumoto et al. (2011)	Medium	Medium	Low	Medium	Medium
McCloud et al. (2004)	High	High	High	High	High
Modestin et al. (2005)	Medium	Medium	Low	Medium	Medium
Pompili et al. (2009)	Medium	Low	High	Medium	Medium
Prigerson et al. (2003)	Medium	High	High	Medium	Medium
Restrepo et al. (2019)	Medium	Medium	Low	Medium	Medium
Salloum et al. (1996)	Medium	Medium	Medium	Medium	Medium
Sher et al. (2007)	Medium	Medium	High	Medium	Medium
Soyka et al. (2001)	Medium	High	High	Medium	Medium
Suppapitiporn (2005)	Medium	Medium	Medium	Medium	Medium
Szerman et al. (2012)	Medium	High	High	High	High
Walsh et al. (2014)	Medium	Low	Low	Low	Low
Yaldizli et al. (2010)	High	High	High	High	High
Youssef et al. (2016)	Low	Low	Low	Low	Low

Grey literature was appraised using the AACODS checklist (Tyndall 2010), which assesses Authority, Accuracy, Coverage, Objectivity, Date and Significance. AACODS scores were expressed as the proportion of items rated positively and are presented in Table 5.

**TABLE 5:** Grey literature and quality ratings.

Document title	Authors/organisation	AACODS score (%)
Coexisting severe mental illness and substance misuse: community health and social care services (2016)	National Institute for Clinical Excellence	100
Annual report 2026: UK patient and general population data 2013–2023	National Confidential Inquiry into Suicide and Safety in Mental Health, The University of Manchester	100
Suicide by people in contact with drug and alcohol services: a national study 2021–2022	National Confidential Inquiry into Suicide and Safety in Mental Health, The University of Manchester	100
Co‐occurring substance use and mental health disorders (2025)	Royal College of Psychiatry	100
Policy paper. Suicide prevention in England: 5‐year cross‐sector strategy. Published 11 September 2023. Suicide prevention in England: 5‐year cross‐sector strategy—GOV.UK	Department of Health and Social Care	100
Dual diagnosis needs assessment: Report prepared for West Sussex County Council 2014	Figure 8 Consultancy	87

### 4.6 Data Extraction and Synthesis

For each empirical study, data were extracted on:

- Study characteristics (author, year, country, design, setting, sample size).
- Definitions and measures of co‐occurring conditions and suicidality.
- Factors associated with co‐occurring conditions and suicidality that were reported by the study authors to be statistically significant.
- Any identified risk or protective factors.

Major findings (those that were reported by the study authors to be statistically significant) were subsequently mapped onto the 5Ps risk‐formulation model: presenting problem, predisposing, precipitating, perpetuating and protective factors (Macneil et al. 2012; Table 6). The 5Ps did not provide the deductive coding framework for data extraction or thematic analysis; rather, it was applied after synthesis to organise the findings in a form familiar to mental health practitioners who use 5Ps formulation in clinical work. Highlight colours in Table 6 indicate the corresponding 5Ps domain, as shown in the key below the table.

**TABLE 6:** Data extraction table: Empirical studies and findings.

First author	Study title	Type of study	Definition of co‐occurring conditions and suicide	Population	Statistically significant findings
Agosti and Levin (2006)	One‐year follow‐up study of suicide attempters treated for drug dependence	Cohort	Diagnostic Interview Schedule, 8 Composite International Diagnostic Interview, 9 and Symptom Checklist 90. 10 Diagnostic Interview Schedule module based on DSM III‐R Suicide = suicide attempt	2228 people admitted to drug treatment centres in the USA	The likelihood of recovery from drug dependence did not differ between patients who did or did not attempt suicide. Among suicide attempters who recovered from substance dependence, the frequency of Major Depression Disorder was significantly lower compared to admission, but its prevalence was nearly three times higher than that found in the general community
Anestis et al. (2012)	The interactive role of distress tolerance and borderline personality disorder in suicide attempts among substance users in residential treatment	Case control	DSM‐IV Borderline personality disorder + SUD Suicide = Lifetime parasuicide count (deliberate self‐harm with intent to die)	176 adults in residential SUD treatment USA, with and without borderline personality disorder	SUD patients with BPD and high distress tolerance are at greatest risk of engaging in chronic and medically serious suicidal behaviour
Blixen et al. (1997)	Dual diagnosis in elders discharged from a psychiatric hospital	Cross sectional	DSM‐III‐R substance abuse diagnosis in addition to a psychiatric diagnosis Suicide = Suicidal ideation and attempts	101 community‐dwelling people aged 65 years and over, discharged to home from three private psychiatric hospitals in the southern USA	No significant differences between the groups in expressed suicidal ideation prior to admission, significantly more people in the dual diagnosis group (17.7%) than in the psychiatric diagnosis group (3.3%) were admitted to the psychiatric hospital for a failed suicide attempt
Carballo et al. (2007)	Pathological personality traits and suicidal ideation among older adolescents and young adults with alcohol misuse: a pilot case–control study in a primary care setting	Case control	DSM‐IV criteria for personality disorder. For alcohol, a positive response to at least 1 item on CAGE screening tool Suicide = Suicidal ideation	42 people aged 18–30 attending a primary care centre	Depressive and anxiety disorders more prevalent among subjects with alcohol misuse than controls. 22.2% of patients with depressive or anxiety disorders and alcohol misuse reported suicidal ideation compared to none among the controls suffering from depressive or anxiety disorders. 69.1% of subjects with alcohol misuse had a comorbid personality disorder. Significantly more adolescents and young adults with alcohol misuse exhibited borderline personality traits (*p* = 0.03) and there was also a trend towards a greater proportion exhibiting histrionic traits (*p* = 0.07) than among those without alcohol misuse
Cornelius et al. (1995)	Disproportionate suicidality in patients with comorbid major depression and alcoholism	Case control	Initial Evaluation Form—validated and consistent with DSM‐III. Suicide = Suicidal ideation, ‘death wishes’, suicidal behaviour	6229 patients seeking evaluation at a USA psychiatric facility	Depressed alcoholics 59% higher suicidality than depressed non‐alcoholics . 22% lower self‐esteem, greater impulsivity , functional impairment and abnormal personal and social history markers
Davis et al. (2005)	Substance use disorder comorbidity in major depressive disorder: a confirmatory analysis of the STAR*D cohort	Case control	Diagnosis of non‐psychotic MDD in DSM‐IV and a score of greater than 14 on the 17‐item Hamilton Rating Scale for Depression Suicide = Suicide attempts	2541 outpatients with major depression (MDD) enrolled in the Sequenced Treatment Alternatives to Relieve Depression study	Compared to those without SUD, MDD patients with concurrent SUD were more likely to be younger, male, divorced or never married, and at greater current suicide risk, and have an earlier age of onset of depression, greater depressive symptomatology, more previous suicide attempts , more frequent concurrent anxiety disorders, and greater functional impairment
De Bernardo et al. (2002)	Comorbid psychiatric and alcohol abuse/dependence disorders: psychosocial stress, abuse, and personal history factors of those in treatment	Case control	DSM‐IV affective disorder + SUD Suicide = Suicide attempts	69 patients at a private rehabilitation unit in USA	Significant differences between dual diagnosis patients and alcohol abuse/dependence only in regard to gender, previous diagnosis, length of illness, suicide attempts , psychotropic medication history , maternal emotional, physical and sexual abuse, paternal abuse, legal difficulties, and psychosocial stressors. No significant differences between substance abusing patients and dually diagnosed patients were found in terms of self‐assessment of progress in treatment
Driessen et al. (1998)	Psychiatric comorbidity, suicidal behaviour and suicidal ideation in alcoholics seeking treatment	Cohort	CIDI, validated DSM/ICD diagnostic criteria used.	149 patients admitted to an alcohol detox facility in Germany	A history of suicide attempts was reported by 29.2% at the outset, and suicidal ideas by 14.1% and suicide attempts by 5.4% at the follow‐up. The following co‐morbidity patterns were associated with the greatest risk for suicidal ideas; Anxiety and depressive disorders, axes I and II disorders, and a history of suicide attempt
Dumais et al. (2005)	Risk factors for suicide completion in major depression: a case–control study of impulsive and aggressive behaviours in men	Case control	DSM‐IV criteria for substance dependence + depression Suicide = Suicide death	104 men who died by suicide who met DSM‐IV criteria for major depressive disorder/depression in Canada	6‐month prevalence of alcohol abuse/dependence, current drug abuse/dependence, and cluster B personality disorders increased the risk of suicide in individuals with major depression . Higher levels of impulsivity and aggression were associated with suicide. Risk factors were more specific to younger suicide victims (ages 18–40). Current alcohol abuse/dependence and cluster B personality disorder were two independent predictors of suicide
Erfan et al. (2010)	Effect of comorbid depression on substance use disorders	Case control	DSM‐IV diagnoses of SUD and depressive disorder Suicide = Ideation and attempt	60 consecutive male inpatients in Egypt	Significant differences (between comorbid depression with SUD and depression only) were found for prescribed opioid use (100% vs. 86%), polysubstance use (73% vs. 43%), frequency of suicidal attempts (86% vs. 43%), mean of admissions to substance use treatment units (5 vs. 2.2), mean of abstinence periods (3.8 vs. 1.2); study group showed greater medical status impairment (*p* = 0.05), social and psychiatric impairment (*p* = 0.01)
Fitzsimons et al. (2007)	Mood disorders affect drug treatment success of drug‐dependent pregnant women	Case control	DSM‐IV criteria for axis 1 disorder + SUD (opiate) Suicide = Suicidal ideation	106 opiate dependent, pregnant women admitted to the Centre for Addiction and Pregnancy (USA) over a 2‐year period	Mood disorder group significantly more likely to be positive for drugs while in treatment compared with both the ‘no disorder’ and ‘anxiety disorder’ groups. MD and AD groups had more psychosocial impairment and higher incidence of suicidal ideation compared with the ND group. AD group spent more days in treatment compared with the ND or MD group
Garlow et al. (2003)	Cocaine use disorders and suicidal ideation	Case control	DSM‐IV criteria Suicide = Suicidal ideation	777 people attending an urban psychiatric emergency service in the USA	More than half of the patients (55.26%) with a substance‐induced mood or psychotic disorder expressed suicidal ideation, and the majority (85%) of these patients had a cocaine use disorder. Cocaine use is associated with suicidal ideation, more so than other substances of abuse
Grant and Hasin (1999)	Suicidal ideation among the United States drinking population: results from the National Longitudinal Alcohol Epidemiologic Survey.	Cohort	DSM‐IV alcohol and drug use disorders and major depression were derived from the Alcohol Use Disorder and Associated Disabilities Interview Schedule Suicide = Suicidal ideation	18 352 current alcohol drinkers in the USA	Past year major depression and alcohol dependence identified as risk factors of suicidal ideation, with major depression having the more sizable impact. Suicidal ideation increased among men with past AUD, and elevated among women who had used drugs nonmedically and developed a drug use disorder during the past year. Occurrence of a recent physical illness and lifetime treatment for major depression among men and women increased the risk of suicidal ideation, whereas marriage was protective against ideation for both sexes. Unemployment and having a family history of alcoholism increased the risk of suicidal ideation among men, but not women
Hakko et al. (2005)	Alcohol‐related suicides in victims with a history of hospital‐treated depression	Cohort	Discharge diagnosis of people treated in hospital for 1. AUD only. 2. Depression only. 3. AUD & depression Suicide = Suicide death	1585 people in Finland who died by suicide with prior hospital admissions	Survival time post hospital discharge for males with AUD and depression is significantly shorter than those with AUD or depression alone
Handley et al. (2016)	Investigation of a Suicide Ideation Risk Profile in People with Co‐occurring Depression and Substance Use Disorder.	Cross sectional	Beck DI above 17. Higher than recommended drinking levels in Australia or cannabis use in excess of once weekly in past month (DAISI and SHADE) Suicide = BDI—2 (assesses severity of suicidal thoughts)	271 people self‐referring to GP/substance use treatment/depression treatment with co‐occurring depression and alcohol misuse taking part in a clinical trial of treatments in Australia	Baseline suicidality is strongest predictor of later (6 months) suicidality—63% at baseline and 43.5% at follow‐up. Younger age of first psychiatric episode increased follow‐up ideation by 16%. Dysfunctional attitudes increased rates of follow‐up ideation by 35%
Harned et al. (2006)	Self‐harm and suicidal behaviour in women with comorbid PTSD and substance dependence.	Cross sectional	Structured Clinical Interview for DSM‐IV Suicide = Suicide attempts, self‐harm, suicidal ideation and self‐harm ideation in last 3 months	65 women outpatients with PTSD and substance dependence in the USA	High rates of suicide attempts, self‐harm, suicidal ideation, and self‐harm ideation in last 3 months. Both PTSD and SD perceived as contributing to self‐harm and suicidal behaviour. Women who had and had not engaged in such behaviour differed in SD diagnoses and reasons for staying safe, but not in the number or type of traumatic experiences, age at first trauma, or substance abuse diagnoses
Landheim et al. (2006)	What characterises substance abusers who commit suicide attempts? Factors related to Axis I disorders and patterns of substance use disorders. A study of treatment‐seeking substance abusers in Norway.	Cross sectional	DSM‐ IV criteria for substance dependence + DSM‐IV axis 1 disorder Suicide = Suicide attempts	256 consecutive attendances at inpatient and outpatient SUD facilities in Norway	Those with an axis 1 disorder were significantly more likely to have attempted suicide. Most significant for major depression, dysthymia, agoraphobia, social phobia, PTSD, eating disorder. GAD not significant
Leposavić et al. (2015)	Comorbidity of harmful use of alcohol in population of schizophrenic patients	Cross sectional	ICD‐10 (MINI) Structured questionnaire for the assessment characteristics and effects of harmful use of alcohol in the population of schizophrenics—modified version Suicide = Suicide attempts	50 inpatients receiving treatment for schizophrenia in Serbia	Significantly higher rate of harmful use of alcohol co‐morbidity in the male population. There is a prominent significant difference in alcoholism heritage in co‐morbidity group. A statistically significant difference between the subgroups was found in the frequency of child neuroticism and adolescent behavioural problems. The results point to a higher suicidal risk and higher rate of cognitive impairment in the co‐morbidity sub‐group
Marín‐Navarrete et al. (2016)	Co‐Occurring Disorders: A Challenge for Mexican Community‐Based Residential Care Facilities for Substance Use	Cross sectional	DSM‐IV criteria for SUD. Mini International Neuropsychiatric Interview–fifth version in Spanish to assess psychiatric conditions Suicide = Thoughts, plans, attempts	601 patients receiving treatment for substance use disorders at 30 facilities located in five Mexican states over 2 years	Co‐occurring disorders associated with higher severity of psychiatric symptoms, more days of substance use, current suicidal ideation, plans, and attempts, more sexual risk behaviours; and more contact with professional services
Matsumoto et al. (2011)	Comparative study of suicide risk in depressive disorder patients with and without problem drinking	Cohort	DSM‐IV diagnosis of depressive disorder Suicide = Suicide risk based on Kessler10 and MINI	917 consecutive outpatients of 5 clinics with depressive disorder in Japan	Female subjects with at least a moderate problem drinking showed significantly more severe depression and suicidality than those without, but no such difference was identified in men
McCloud et al. (2004)	Relationship between alcohol use disorders and suicidality in a psychiatric population: in‐patient prevalence study.	Cross sectional	Self‐completed lifestyle survey, AUDIT. Substance Abuse Assessment Questionnaire. ICD‐10 diagnosis. Suicide = Records indicate they had carried out any self‐harm/had had thoughts of self‐harm/suicide	200 consecutive psychiatric admissions in the UK	Out of 200 subjects, 48.5% scored 8 or more (indicating hazardous or harmful alcohol use) and 22.5% scored 16 or more (indicating significant alcohol dependence) on the AUDIT. There were no significant gender differences. Alcohol misuse was strongly associated with suicidality
Modestin et al. (2005)	Two types of classification for male opioid dependence: identification of an opioid addict with depressive features.	Cross sectional	Extended semi‐structured clinical interview, considering four classification criteria sets devised by Cloninger et al. (1981), von Knorring et al. (1985, 1987), Buydens‐Branchey (1989) and Babor et al. (1992). DSM‐IV mental health diagnostic criteria Suicide = Severe suicide attempts	100 hospitalised adult opioid dependent men in Switzerland	The C2 patient is younger, has a history of therapy because of depression and a history of severe suicide attempts, also abuses benzodiazepines and becomes violent while intoxicated . His father suffers from alcoholism and received treatment because of depression. The C1 patient lacks these characteristics
Pompili et al. (2009)	Substance abuse, temperament and suicide risk: evidence from a case–control study.	Case control	DSM‐IV diagnosis of substance abuse Suicide = Suicidal ideation	62 consecutive psychiatric outpatients in Rome	Substance abusers had higher dysthymia, cyclothymia and anxiety trait scores. Severity of psychopathology/psychological problems. Higher lifetime suicidal ideation (strongest association)
Prigerson et al. (2003)	Suicidal ideation and suicide attempts in homeless mentally ill persons: Age‐specific risks of substance abuse	Case control	30‐item algorithm. Self‐report scales for symptoms of depression, psychosis, and mania, structured interviewer observations of overtly disturbed behaviour. Self‐reported number of days drinking to intoxication in previous 30 days and total number of days of illegal drug use in the prior 30 days using items from The Addiction Severity Index Suicide = Suicidal ideation and attempts	7224 people homeless for at least 7 of last 14 days with evidence of a serious mental illness in the USA	Prevalence of 30‐day suicidal ideation and suicide attempts (37.5% and 7.9%, respectively) was extremely high. Risk of serious suicidal ideation and suicide attempts greater among the younger compared with the older homeless mentally ill clients, risks were not significantly increased by co‐morbid alcohol and/or drug abuse. A significant interaction between age and comorbid substance abuse was observed showing that among older clients but not younger clients, those with drug and alcohol abuse were at significantly greater risk of suicidal ideation than those without substance use problems
Restrepo et al. (2019)	Suicide Risk Associated with Dual Diagnosis in General Population.	Cross‐sectional	CIDI 3.0 CAPI highly structured interview designed by WHO with Drug & Alcohol Administration and Mental Health Administration for use by lay reviewers uses DSM‐IV diagnoses Suicide = ideas, plans, attempts	303 adults from the general population of a city in Columbia	A strong association was found between suicidality and dual diagnosis
Salloum et al. (1996)	Disproportionate lethality in psychiatric patients with concurrent alcohol and cocaine abuse.	Cross sectional	DSM‐III criteria for axis 1 and SUD Suicide = Suicidal ideation and plans	101 patients consecutively admitted to a specialist dual diagnosis unit in the USA	Alcohol and cocaine abuse group had a higher likelihood of associated current homicidal behaviour than the alcohol‐only and the cocaine‐only groups
Sher et al. (2007)	Triggers for suicidal behaviour in depressed older adolescents and young adults: do alcohol use disorders make a difference?	Case control	DSM‐IV diagnosis using Structured Clinical Interview (SCID‐I) Depression and suicide attempt with or without co‐occurring alcohol abuse or dependence Suicide = Suicide attempt	67 people aged 18–26 with depression who had attempted suicide in the USA	Depressed suicide attempters with alcohol use disorders had higher aggression and impulsivity scores and tended towards higher lethality attempts
Soyka et al. (2001)	Psychopathology in dual diagnosis and non‐addicted schizophrenics: Are there differences?	Cross sectional	ICD‐9 diagnosis of schizophrenia. Munich alcoholism screening test, reported use of drugs Suicide = Suicide attempts	637 consecutive patients with schizophrenia admitted to psychiatric hospital in Germany	Dual diagnosis patients had more previous suicide attempts and more often a history of delinquency
Suppapitiporn (2005)	Comorbidity of alcohol dependence in suicidal depressed patients.	Cohort	Clinical diagnostic interview including Hamilton rating scale for depression (HAM‐D). DSM‐IV for substance‐use disorder/dependence Suicide = Suicide attempt	110 patients admitted to psychiatric hospital (over the course of 12 months) in Thailand following a suicide attempt who were assessed to be clinically depressed	Factors associated with suicidal depressed patients who had comorbid alcohol use disorder were male, aged above 30 years and lower education and additionally, in those with alcohol dependence were associated with stressor about financial problems and less likely to attempt suicide by acetaminophen overdose or be precipitated by relationship problem
Szerman et al. (2012)	Dual diagnosis and suicide risk in a Spanish outpatient sample.	Case control	Presence of any SUD and any other Axis 1 diagnosis according to the MINI or Axis 2 according to the PDQ4+ (ICD‐10) Suicide = Suicide risk scale (screening tool not specified)	837 consecutive patients seeking outpatient treatment at either for their mental health, substance use, or both, in Spain	Increased suicide risk associated with DD
Walsh et al. (2014)	Characteristics of Immigrant and Non‐Immigrant Patients in a Dual‐Diagnosis Psychiatric Ward and Treatment Implications	Case control	ICD‐10 criteria for dual diagnosis (axis 1 disorder + substance use) Suicide = Suicide attempts	554 patients hospitalised in a male dual diagnosis ward of a community psychiatric hospital in Israel over a 5‐year period	Non‐immigrant patients reported higher numbers of repeat and involuntary hospitalisations and more drug use. Immigrants showed longer hospitalisations , more suicide attempts, more violent suicide attempts, more violent offences and more alcohol use. Among non‐immigrants significant relationships were found between severity of SMI and crime/violence while among immigrants a significant relationship was found between suicidality and crime/violence
Yaldizli et al. (2010)	Risk factors for suicide attempts in patients with alcohol dependence or abuse and a history of depressive symptoms: A subgroup analysis from the WHO/ISBRA study	Cross sectional	Extensive interview instrument developed with U.S. National Institute on Alcohol Abuse and Alcoholism including psychiatric evaluation. DSM‐IV criteria for depression Suicide = Suicide attempt	1863 multi‐national (participants from Brazil, Australia, Finland, Japan and Canada) from the general population	Drinking status, adverse experiences during drinking, bad experiences from drug abuse, depressive symptoms and antidepressant treatment are independent risk factors for suicide attempt
Youssef et al. (2016)	Dual Diagnosis and Suicide Probability in Poly‐Drug Users	Case control	Psychiatric review against DSM‐IV criteria (SUD and mental illness) Suicide = Lifetime history of ideation or attempt	117 male adults admitted to a mental health hospital in Saudi Arabia with poly‐substance use	Higher rates of mental illness were found in the suicidal group (substance induced mood disorder, schizoaffective disorder, major depressive disorder, antisocial and borderline personality disorder)

*Note:* Key: Green, Prevalence; Blue, presenting problem; Yellow, predisposing factor; Pink, precipitating factor; Purple, perpetuating factor; Red, protective factor; Grey, patient experience.

During this process, a sixth domain, Patient Experience, emerged inductively to capture issues relating to access to care, service design, and experiences of stigma or exclusion.

Finally, the synthesised findings were interpreted through the lens of the Integrated Motivational–Volitional (IMV) Model of suicidal behaviour (O'Connor 2011; O'Connor and Kirtley 2018), focusing on how identified factors might contribute to pre‐motivational, motivational and volitional phases of suicide risk.

## 5 Results

### 5.1 Overview of Included Studies

The 33 empirical studies involved 35 883 participants across 19 countries, including the United States, United Kingdom, European nations, Australia, Latin America, the Middle East and Asia. Mental health diagnoses included mood, psychotic, anxiety, trauma‐related, personality and eating disorders, defined using DSM or ICD criteria, whereas substance use disorders (SUDs) included alcohol, opioids, stimulants and poly‐substance use.

All empirical studies were quantitative; there were no qualitative studies identified relating to suicidality in people with co‐occurring conditions. Whilst surprising, this is not believed to be related to study search criteria or exclusion criteria, as academic database searches were cross‐checked against internet searches and discussions with researchers and clinicians. Overall study quality was generally medium, with some high‐quality studies of mood disorders and alcohol use.

Six grey literature sources included national guidelines (NICE 2016), policy and strategy documents (DHSC 2023; Public Health England 2017), national suicide audits (NCISH 2024, 2025), and a Royal College of Psychiatrists report on co‐occurring conditions (Royal College of Psychiatrists 2025). AACODS scores were high (87%–100%).

To support clinical application, the findings were mapped onto the 5Ps risk formulation model. Many studies compared suicidality among people with co‐occurring conditions with suicidality among those who had either a mental health condition or a SUD alone. A sixth category, ‘Patient Experience’, was added to capture how people with co‐occurring conditions experience services, including barriers to accessing services and the effects of service design, which may compound suicide risk.

#### 5.1.1 Prevalence of Suicidality in People With Co‐Occurring Conditions

Twenty studies examined the association between co‐occurring conditions and suicidality. Across these, people with co‐occurring conditions had higher rates of suicidality than those with either a mental health diagnosis alone or a SUD alone. Study quality was rated low in three, medium in 13, and high in four (Carballo et al. 2007; De Bernardo et al. 2002; Youssef et al. 2016; Blixen et al. 1997; Cornelius et al. 1995; Erfan et al. 2010; Fitzsimons et al. 2007; Garlow et al. 2003; Grant and Hasin 1999; Hakko et al. 2005; Harned et al. 2006; Marín‐Navarrete et al. 2016; Matsumoto et al. 2011; Pompili et al. 2009; Restrepo et al. 2019; Soyka et al. 2001; Davis et al. 2006; Dumais et al. 2005; McCloud et al. 2004; Szerman et al. 2012). One medium‐quality study reported increased suicidality in women but not men (Matsumoto et al. 2011).

In the grey literature, NCISH (2026) reports that among those who died by suicide in the United Kingdom between 2013 and 2023, 46% had alcohol misuse and 38% had drug misuse (people who were using both alcohol and drugs will be represented in both percentages). Among mental health patients who died by suicide, 56% had a history of alcohol or drug use. Although these data are not restricted to people with diagnosed co‐occurring conditions, they are consistent with increased risk in this group.

Overall, the evidence strongly indicates increased suicidality in people with co‐occurring conditions compared to those with a mental health condition or SUD alone.

Table 7 summarises factors associated with increased suicide risk, grouped into prevalence, presenting problem, predisposing, precipitating, perpetuating, protective factors and patient experience.

**TABLE 7:** Factors highlighted as more prevalent/severe in people with co‐occurring conditions presented within the 5Ps model.

Risk categories	Factors associated with increased suicide risk in people with co‐occurring conditions	Data source (E = empirical study, G = grey literature)
Presenting problem: This is a detailed description of the difficulties the individual is currently experiencing. It includes their thoughts, feelings, and behaviours	Symptom severity + Attitudes and beliefs Functional impairment +	E E E
Predisposing factors: Historical and background factors that can make someone more vulnerable to mental health issues, including genetics, early life experiences, or other long‐term vulnerabilities	Mental health diagnosis Earlier age of onset of mental health symptoms Substance use disorder Physical ill health/disability + Psychological development Adverse childhood experiences +	E, G E E, G E, G E E, G
Precipitating factors: Immediate triggers or events that seem to have led to the onset of the problem. Examples include a loss of a loved one, a major life change, or a significant stressor	Child removal Unemployment Financial problems Relationship problems Legal problems	E, G G E E, G E, G
Perpetuating factors: These are the factors that keep the problem going. They can be ongoing unhelpful coping strategies, substance use, or recurring patterns of thought or behaviour	Impulsivity + Distress tolerance + Aggression +	E, G E, G E
Protective factors: These are the internal and external strengths and resources that help an individual cope and maintain their mental well‐being. They can be personal traits, supportive relationships, or activities like hobbies	Marriage/partnership Parental responsibility Belief in ability to cope	E E E
Patient experience: This is a proposed sixth category relating ability to access services, service design and policy and the experience of patients when accessing mental health services	Access to mental health services − Access to drug and alcohol services − Dissatisfaction with services + History of mental health treatment More days in treatment	G G G E E

## 6 The 5Ps Model of Risk Formulation

### 6.1 Presenting Problem

Within the 5Ps, the presenting problem describes the current difficulties, including thoughts, feelings, and behaviours.

#### 6.1.1 Symptom Severity

There is strong evidence that people with co‐occurring conditions experience greater mental health symptom severity associated with higher suicidality. Five studies (one high, four medium quality) reported links between co‐occurring conditions, suicidality and increased severity of mental health symptoms, including functional and psychiatric impairment and broader psychological problems (Davis et al. 2005; Marín‐Navarrete et al. 2016; Cornelius et al. 1995; Erfan et al. 2010; Pompili et al. 2009). The Royal College of Psychiatrists (Royal College of Psychiatrists 2025) further notes that prolonged heavy drinking is associated with increased risk of depression, anxiety, self‐harm and suicide and a longer and more severe course of bipolar disorder.

#### 6.1.2 Functional Impairment

Five studies (one high, four medium quality) reported greater functional impairment among people with co‐occurring conditions across medical, social, psychiatric, psychosocial and cognitive domains (Davis et al. 2006; Cornelius et al. 1995; Erfan et al. 2010; Fitzsimons et al. 2007; Leposavić et al. 2015). This suggests more substantial challenges in day‐to‐day functioning.

#### 6.1.3 Attitudes and Beliefs

Handley et al. (2016) (high quality) found that among people with co‐occurring depression and SUD, dysfunctional attitudes increased follow‐up suicidal ideation by 35%. Dysfunctional attitudes were measured with the Weissman and Beck (1978) scale, which explores beliefs about achievement, dependence on others, and self‐control. Cornelius et al. (1995) (medium quality) reported that lower self‐esteem was associated with suicidality in people with co‐occurring alcohol use disorder (AUD) and depression.

### 6.2 Predisposing Factors

Predisposing factors are historical or background influences that increase vulnerability, such as genetics, early experiences or chronic adversity. In co‐occurring conditions, these include mental health diagnosis, age of onset, SUD, physical illness, psychological development and adverse childhood experiences.

#### 6.2.1 Mental Health Diagnosis

Mood disorders, especially depression, are common and highly relevant. Globally, around 4% of the population experience a depressive disorder in any year (Dattani et al. 2023). In the United Kingdom, mood disorders accounted for 39% of mental health patient suicides between 2012 and 2022 (NCISH 2025). Twelve of the 33 empirical studies focused primarily on mood disorders.

Three medium‐quality studies reported that suicidality was 43%–59% higher among people with co‐occurring depression and SUD than in those with depression alone or SUD alone (Cornelius et al. 1995; Grant and Hasin 1999; Erfan et al. 2010). Four studies (one low, three medium quality) examined SUD effects in heterogeneous mental health populations and found the strongest association with suicidality in mood disorders, particularly depression; significant associations were also reported for anxiety, trauma‐ and stress‐related disorders, eating disorders, personality disorders and psychotic disorders (Driessen et al. 1998; Youssef et al. 2016; Landheim et al. 2006; Pompili et al. 2009).

Salloum et al. (1996) (medium quality) reported that in psychiatric patients with alcohol, cocaine or combined alcohol–cocaine use, co‐occurring depression was associated with higher rates of suicidal plans in the combined‐use group. Two medium‐quality studies in schizophrenia found higher suicidality associated with SUD: Soyka et al. (2001) reported a 12.7% increase in suicide attempts, and Leposavić et al. (2015) found a 38% increase in suicidal attempts and statements in patients with schizophrenia and AUD.

NCISH (2024) reported that among people who died by suicide and had been in contact with drug and alcohol services, 30% had also seen mental health services; the most common diagnoses were affective disorders and personality disorders. DHSC (2023) reports that both acute intoxication and dependence on alcohol and/or drugs are consistently associated with substantially increased risk of suicide and self‐harm.

Taken together, these findings support a strong association between mood disorders (particularly depression) and elevated suicide risk in people with co‐occurring conditions, with additional contribution from other mental disorders when combined with SUD.

#### 6.2.2 Age of Onset of Mental Health Problems

Three studies (two high, one low quality) found that earlier onset of mental health conditions was associated with increased suicidality among people with co‐occurring mood and substance use disorders (Handley et al. 2016; Davis et al. 2005; De Bernardo et al. 2002).

#### 6.2.3 Substance Use Disorder

Twelve studies focused on AUD, making it the most frequently investigated SUD. AUD is also the most common SUD worldwide, with an estimated 7% of adults meeting AUD criteria in 2019 (WHO 2024).

Findings for substance type were mixed. Agosti and Levin (2006) (medium quality) found no clear association between suicidality and specific substances. Dumais et al. (2005) (high quality) found higher rates of AUD and other SUDs among men with depression who died by suicide, with both contributing independently to risk. Garlow et al. (2003) (medium quality) reported that 55% of people with substance‐induced mood or psychotic disorders expressed suicidal ideation, 85% of whom had a cocaine use disorder.

In women with PTSD and substance dependence, alcohol and poly‐substance dependence (but not opiate dependence) were more common among those with recent self‐harm or suicide attempts (Harned et al. 2006). Salloum et al. (1996) found very high rates of suicidality across alcohol, cocaine, and combined use, with no major differences by substance pattern.

Two studies, one high quality and one medium quality, found that greater SUD severity in people with co‐occurring conditions was associated with increased suicidality (Marín‐Navarrete et al. 2016; McCloud et al. 2004). Erfan et al. (2010), a medium quality study, reported that use of multiple substances was associated with higher suicide risk, more treatment admissions, and shorter periods of abstinence.

#### 6.2.4 Physical Ill Health/Disability

Physical illness and impairment were associated with suicidal ideation in two medium‐quality studies (Grant and Hasin 1999; Erfan et al. 2010). NCISH (2024) found that people who died by suicide within 12 months of contact with drug and alcohol services were more likely to experience physical ill health.

#### 6.2.5 Psychological Development

Two medium‐quality studies reported higher rates of historical delinquency, adolescent behavioural problems and childhood neuroticism among people with schizophrenia and co‐occurring SUD (Soyka et al. 2001; Leposavić et al. 2015).

#### 6.2.6 Adverse Childhood Experiences

Two medium‐quality and one low‐quality study reported higher levels of adverse experiences in people with co‐occurring conditions, including ‘broken’ families, academic difficulties, behavioural problems, perinatal problems, developmental delays, family history of alcoholism, and maternal and paternal abuse (Cornelius et al. 1995; Grant and Hasin 1999; De Bernardo et al. 2002).

### 6.3 Precipitating Factors

Precipitating factors are immediate events or stressors that appear to trigger the onset or escalation of problems.

#### 6.3.1 Child Removal

Royal College of Psychiatrists (2025) report that women facing, or following, child removal are at higher risk of relapse and suicide, and that engagement with services may be disrupted. Some services withdraw when parental responsibility is lost. Although parental responsibility can be protective, loss of custody may function as a potent precipitant of suicide risk.

#### 6.3.2 Unemployment, Financial, Relationship and Legal Difficulties

Suppapitiporn (2005) (medium quality) identified financial and relationship problems as social stressors associated with suicide attempts in people with co‐occurring alcohol dependence and depression. De Bernardo et al. (2002) (low quality) reported that people with co‐occurring conditions were more likely to experience legal problems related to substance use and primary support, social, environmental and occupational difficulties. Erfan et al. (2010) (medium quality) found greater social impairment among those with co‐occurring depression and SUD. Two studies (one high, one medium quality) linked increased suicidality to being divorced or never married (Davis et al. 2005; Grant and Hasin 1999).

NCISH (2024) found that people who died by suicide within 12 months of contact with drug and alcohol services frequently experienced socio‐economic adversity, interpersonal problems and physical ill health. DHSC (2023), citing NCISH (2017), reports that 49% of middle‐aged men who died by suicide had alcohol misuse, drug misuse or both, particularly where they were unemployed, bereaved or had a history of self‐harm or violence.

#### 6.3.3 Sex

Davis et al. (2006), a high‐quality study, and Suppapitiporn (2005), a medium‐quality study, reported higher suicidality among men than women with co‐occurring conditions. In contrast, Matsumoto et al. (2011), a medium‐quality study, found higher suicide‐risk scores among women with moderate or severe alcohol problems, whereas scores differed little between men with no alcohol problem and those with moderate or severe alcohol problems. This finding appears inconsistent with the broader mortality pattern reported by NCISH (2025), in which men accounted for 64% of suicides among mental health patients and 76% among people in contact with drug and alcohol services. However, these mortality data do not directly establish the relative effect of co‐occurring conditions by sex. The available evidence is therefore insufficient to support firm conclusions about sex‐specific patterns beyond the general finding of higher suicide mortality among men.

#### 6.3.4 Age

Two medium‐quality studies (Suppapitiporn 2005; Prigerson et al. 2003) found increased suicidality among older participants (defined as > 30 and > 55, respectively) with co‐occurring conditions. NCISH (2024) reported that 61% of suicide deaths among people in contact with drug and alcohol services occurred between ages 35 and 54, roughly mirroring the age distribution of the treatment population. As with sex, the available data are insufficient to draw definitive conclusions regarding age.

### 6.4 Perpetuating Factors

Perpetuating factors maintain or exacerbate difficulties over time. For co‐occurring conditions, these include impulsivity, distress tolerance, and aggression; substance use itself is typically treated as a perpetuating factor but may also be predisposing.

#### 6.4.1 Impulsivity

Three studies (one high, two medium quality) reported heightened impulsivity in people with co‐occurring conditions associated with suicidality (Dumais et al. 2005; Cornelius et al. 1995; Sher et al. 2007).

#### 6.4.2 High Distress Tolerance

Anestis et al. (2012), a medium quality study, found that among people with borderline personality disorder and SUD, those with high distress tolerance were at greatest risk of chronic and medically serious suicidal behaviour. Higher distress tolerance has also been associated with greater acquired capability for suicide (Anestis et al. 2012). Although this may appear counterintuitive because suicide is often associated with low distress tolerance and a desire to escape distress, Joiner (2005) proposed that habituation to physiological pain and fear of death is necessary for a person with suicidal thoughts to engage in suicidal acts. In other words, suicidal behaviour is physically painful and frightening; engaging in serious suicidal behaviour may therefore require a person to prioritise death over the desire for an immediate reduction in physical or emotional pain.

#### 6.4.3 Aggression

Five studies (one high, three medium, one low quality) linked aggression or violence to suicidality among people with co‐occurring conditions (Dumais et al. 2005; Modestin et al. 2005; Salloum et al. 1996; Sher et al. 2007; Walsh et al. 2014).

### 6.5 Protective Factors

Evidence on protective factors is limited. Harned et al. (2006) (medium quality) reported that, among women with PTSD and co‐occurring SUD, concerns about children, a wish to survive and belief in personal coping ability were associated with lower suicidal behaviour. Grant and Hasin (1999) (medium quality) identified marriage as a protective factor, whereas Davis et al. (2006) (high quality) found that being divorced or never married was associated with increased suicide risk.

### 6.6 Patient Experience

#### 6.6.1 Access to Mental Health Services

Six studies (four medium, two low quality) found associations between past mental health treatment and suicidality. Two medium‐quality studies linked higher suicide risk with having received treatment for depression (Grant and Hasin 1999; Modestin et al. 2005). Marín‐Navarrete et al. (2016) (medium quality) reported that people with co‐occurring conditions and suicidality had more contact with professional services, and Fitzsimons et al. (2007) (medium quality) found they spent more days in treatment. Walsh et al. (2014) (low quality) reported more frequent involuntary and longer hospitalisations in this group. However, these studies do not explore patients' subjective experiences or the quality of care received.

Royal College of Psychiatrists (2025) highlight ongoing difficulties in accessing care for people with co‐occurring conditions, including when individuals seek urgent help for suicidal thoughts. Access problems are not explicitly identified as a risk factor in the empirical studies but are prominent in policy and practice literature. NCISH (2024) report that among those who died by suicide within 12 months of contact with both drug and alcohol and mental health services, 20% missed their last mental health contact and 14% were non‐adherent with prescribed medication.

#### 6.6.2 Access to Drug and Alcohol Services

NCISH (2025) report that despite the high prevalence of substance use, only a minority of patients who died by suicide were in contact with specialist alcohol and drug services. In England, 54% of mental health patients who died by suicide had a history of SUD, but only 11% had been in contact with substance use treatment services between 2012 and 2014 (NCISH, as reported by RCPsych 2025). This suggests limited access to, or engagement with, drug and alcohol services among high‐risk individuals.

The West Sussex Dual Diagnosis Joint Strategic Needs Assessment (Figure 8 Consultancy 2014) includes personal accounts from people with co‐occurring conditions supported by a homelessness charity, two of which explicitly describe suicidality. These accounts highlight extensive childhood trauma, long‐term mental health problems, substance use, and pronounced dissatisfaction with mental health services, including rejection by services and a reliance on medication without psychological therapies.

## 7 Discussion

This ILR aimed to synthesise the evidence on suicidality in adults with co‐occurring substance use and mental health conditions, identify risk and protective factors, and apply these findings to refine risk formulation approaches commonly used in mental health nursing. The findings confirm that adults with co‐occurring substance use and mental health conditions are at substantially higher risk of suicidality than those with either condition alone. This was true for every studied combination of a mental health condition and a substance use disorder, although the strongest association with suicide was found in people with co‐occurring mood disorders and AUD. The high prevalence found in the review is important; however, identifying risk requires a fuller understanding of the interaction among the multiple factors identified. The increased risk appears to result from:

- Greater severity of psychiatric symptoms and functional impairment.
- Accumulated adversity across the life course (including adverse childhood experiences, early‐onset mental illness, physical illness and social marginalisation).
- Ongoing exposure to socio‐economic and relational stressors.
- Perpetuating factors such as impulsivity, high distress tolerance, aggression and continued substance use.
- Limited protective factors.
- Service‐related factors, including difficulties accessing care and experiences of stigma and exclusion.

Mapping the findings onto the 5Ps model demonstrates that people with co‐occurring conditions are more likely to present with increased suicide risk factors across all domains of the 5Ps model. Yet in the 5Ps model presented by Macneil et al. (2012), co‐occurring substance use is documented only within the Perpetuating factors section citing its role as a barrier to engagement or adherence to mental health treatment. This underestimates the life experiences and circumstances that lead to substance use disorder and the impact of these on the severity of presentation, acute stressors, and the availability of protective resources uncovered within this ILR which improves understanding of the aetiology of suicidality in people with co‐occurring conditions. Closer attention needs to be paid to the full range of risk factors in people with co‐occurring conditions.

This elevated risk may be further compounded by the challenges that people with co‐occurring conditions face in accessing effective mental health treatment, as identified by the Royal College of Psychiatrists (2025) and the Department of Health and Social Care. The proposed sixth P, Patient experience, has been added on the basis of findings about the challenges that people with co‐occurring conditions face when accessing services. These findings are supported by a range of other studies highlighting staff attitudes and institutional processes that negatively affect this patient group. Evidence indicates that stigma towards this patient group has remained prevalent within health services until recent times. A study involving interviews with people who had lived experience of co‐occurring conditions identified inadequacies in service provision, including the negative effects of exclusion and stigma (Lawrence‐Jones 2010). A survey examining psychiatrists' attitudes towards patients with co‐occurring conditions found that stigmatising attitudes were prevalent (Avery et al. 2013). In addition, a systematic review of mental health nurses' attitudes towards patients with co‐occurring conditions found that a high proportion of nurses held negative beliefs, such as viewing SUD as a self‐inflicted choice, and lacked willingness to care for this patient group or held pessimistic views about treatment effectiveness (Merrick et al. 2022). A study based on interviews with mental health nurses found that their colleagues lacked enthusiasm for working with people with co‐occurring conditions and held harmful attitudes towards this patient group, which can contribute to feelings of helplessness among patients (Anandan et al. 2024). Despite a national policy focus on co‐occurring conditions, people continue to face barriers to accessing mental health treatment and may experience negative staff attitudes when they obtain access (Department of Health and Social Care/NHS England 2025). Previous experiences of being stigmatised by services can affect people's willingness to be referred for treatment, creating additional barriers to effective care (Knaak et al. 2017). Being told that one cannot be helped by mental health services may increase feelings of hopelessness, which have been identified as an important predictor of suicide (Tonkuş et al. 2022). In summary, stigma is prevalent, and its effects include both creating additional barriers to treatment for people with co‐occurring conditions and contributing to psychological processes associated with suicide. Considering a person's past experiences with mental health services as part of their suicide risk formulation could therefore play an important role in understanding their suicide risk, which is why an additional P, Patient experience, is proposed.

There was limited evidence on protective factors within the studies reviewed; having children, having a strong wish to survive, believing in one's ability to cope, and being married were the only factors identified as protective against suicide for people with co‐occurring conditions (Harned et al. 2006; Grant and Hasin 1999). Although protective factors reduce the risk of suicide, the identified factors are less likely to be present in patients with co‐occurring conditions. There is also evidence suggesting that a lack of protective factors may increase suicidality. In the UK, mental health and substance use account for a large proportion of the reasons for statutory assessments of children's welfare: mental health is a factor in 36.6% of assessments, drug use in 19.3%, and alcohol use in 18.4% (Public Health England 2018). Wall‐Wieler et al. (2018) found that mothers whose child had been removed from their custody were significantly more likely to attempt or complete suicide. Regarding marriage as a protective factor, rates of marital dissolution and divorce have been found to be significantly higher among people with AUD (Cranford 2014), and substance use is one of the most commonly cited reasons for divorce (Scott 2013). Men have been found to have higher rates of suicide attempts and completion in the immediate aftermath of a relationship breakdown, as well as higher rates of suicidality in the longer term when divorced or separated (Wilson et al. 2025). Poor coping ability has been identified as a risk factor for suicide (John and Gross 2004), and people with major depression and substance use disorder have been found to have higher maladaptive coping‐strategy scores (Adan et al. 2017). Therefore, the protective factors identified in the reviewed papers are less likely to be present in people with co‐occurring conditions, and their absence is associated with an increased risk of suicide. It is therefore important to use the protective factors element of a 5Ps formulation to highlight that the absence or recent loss of a protective factor is associated with elevated risk.

There was limited exploration of psychological risk factors within the reviewed studies; this has also been a criticism of the application of the 5Ps model. Judd (2020) critiques the 5Ps model as reductive, arguing that it highlights objective risk factors while neglecting a person's subjective lived experience and advocating for a phenomenological approach that prioritises understanding the meaning of that experience. Another critic of the 5Ps model is Pompili (2020), who identified that clinicians too often pay little attention to the phenomenology of the suicidal mind and recommended that exploring mental pain, as the main ingredient of suicide, remains a central unmet need in suicide prevention. Indeed, few of the studies within this ILR focused on mental pain and its expression. The Integrated Motivational‐Volitional Model of Suicidal Behaviour (IMV), proposed by O'Connor (2011) and refined by O'Connor and Kirtley (2018), highlights the importance of incorporating the psychological processes underlying suicidal ideation and the dynamic nature of its progression into action. The authors describe feelings of defeat and humiliation, thwarted belongingness and burdensomeness as key to the development of suicidal ideas and the progression from ideas to plans and actions. The sense of defeat described within the IMV model could be compared with self‐condemnation arising from shame, guilt and self‐stigma, which has been shown to be associated with suicide and substance use issues (Webb and Boye 2024). Thwarted belongingness and a sense of being a burden to others are identified as psychological processes related to suicide and have been found to be more common among people who use multiple substances (Silva et al. 2015). In a study of people with problematic alcohol use, perceived burdensomeness was found to have a significant association with suicidal ideation (Nichols et al. 2022). There is an identified need to better incorporate psychological processes within the 5Ps model, and this may be particularly pertinent for this patient group.

An updated version of the 5Ps model is required to better account for the psychological processes associated with suicide, highlight the increased suicide risk associated with a lack or loss of protective factors, and explore the patient's experience of accessing mental health services. All of these factors are associated with increased suicide risk in people with co‐occurring conditions. The model may also have utility in the wider mental health population, not only among those with co‐occurring conditions (see Table 8).

**TABLE 8:** The updated ‘6Ps’ model of formulation (additions to model are presented in bold).

Risk categories	Description
Presenting problem	This is a detailed description of the difficulties that the individual is currently experiencing, including their degree of severity. It includes the individual's thoughts, feelings and behaviours, **with a strong focus on psychological processes such as a sense of defeat, entrapment, ‘burdensomeness’ and thwarted belongingness. Problematic drug and alcohol use should also be documented**
Predisposing factors	Historical and background factors that can make someone more vulnerable to mental health issues, including genetics, early life experiences, or other long‐term vulnerabilities
Precipitating factors	Immediate triggers or events that seem to have led to the onset of the problem. Examples include a loss of a loved one, a major life change, or a significant stressor
Perpetuating factors	These are the factors that keep the problem going. They can be ongoing unhelpful coping strategies, substance use, or recurring patterns of thought or behaviour
Protective factors	These are the internal and external strengths and resources that help an individual cope and maintain their mental well‐being. They can be personal traits, supportive relationships, or activities like hobbies. **Attention should be paid to the effect of a lack or loss of protective factors in increasing suicide risk**
**Patient experience**	**This domain covers the ability to access services, service design and policy, and experiences of stigma and discrimination when accessing mental health services**

### 7.1 Clinical Practice Implications for Mental Health Nurses and Other Professionals

Key implications include:

- *Prioritising co‐occurring conditions as high risk*: People with co‐occurring conditions should not be regarded as “too complex” or deferred until substance use is stabilised. Instead, their combined risk profile warrants proactive mental health intervention.
- *Using an extended 6Ps framework*: Mental health professionals can use the 5Ps plus Patient Experience to structure comprehensive suicide risk formulations
- *Attending to patient experience*: Mental health professionals should specifically ask about experiences of seeking help (e.g., being turned away, feeling blamed or stigmatised) and explore how these experiences influence current hope and trust in services.
- *Integrating psychological processes*: Routine assessment should include constructs such as hopelessness, mental pain, and perceived burdensomeness, informed by the IMV model, rather than focusing solely on historic risk factors.
- *Recognising the effect of absent or lost Protective factors on suicide risk*: Mental health professionals should understand that people with co‐occurring conditions are more likely to lack or have lost Protective factors and that this absence or loss can increase suicide risk.

### 7.2 Strengths and Limitations of the Review

The use of ILR methodology has enabled the review to capture diverse evidence relating to a complex clinical problem, providing a richer set of results. The systematic search across major health databases and the structured appraisal using the CASP and AACODS tools provide methodological rigour and ensure replicability. Overlaying the findings onto a widely used clinical framework (the 5Ps) has enabled the generation of practice‐relevant recommendations for mental health nurses and other mental health professionals.

A limitation of this ILR is the lack of resources to enable 100% of the articles to be appraised independently by two reviewers. Measures were taken to promote reliability and consistency, including the use of highly detailed, recognised appraisal tools and checks of inter‐rater reliability. Another limitation is that all the empirical studies were quantitative and observational. No qualitative studies relating to suicidality in people with co‐occurring conditions were found, limiting insight into lived experiences and mechanisms. Heterogeneity across studies complicated direct comparisons. The studies included a variety of mental health diagnoses and substances used, and it is difficult to argue that the same findings apply across such a diverse group of patients. However, the findings indicated that risk increases regardless of the specific diagnosis or substance, suggesting that there is something about their co‐occurrence that increases risk and warrants closer attention. Most studies were conducted in specialist services or subgroups, such as those defined by specific diagnoses or substances, which may limit generalisability. Causal inferences cannot be drawn, and the findings indicate associations rather than the direction of effects.

### 7.3 Future Research

Further research is needed to examine how mental health professionals currently assess and formulate suicide risk in people with co‐occurring conditions. Understanding what influences professionals' knowledge and practice when formulating suicide risk for people with co‐occurring conditions will help to determine how practice can best be improved. Qualitative exploration is required of how mental health professionals elicit and describe the psychological processes (e.g., hopelessness, mental pain and perceived burdensomeness) and patient experiences that shape risk. Consideration should also be given to evaluating training interventions and the effects of organisational changes that promote integrated care, reduce stigma and support formulation‐based practice. The development and testing of risk‐formulation tools and templates that operationalise the extended 5Ps plus Patient experience model for routine practice would be valuable.

## 8 Conclusion

This ILR is, to our knowledge, the first to synthesise evidence on suicidality in people with co‐occurring substance use and mental health conditions and to explicitly map findings onto a risk formulation framework used in mental health services. Co‐occurring conditions substantially increase suicide risk and are characterised by multiple, interacting risk factors across the life course, few identified protective factors, and significant barriers to effective care.

For mental health nurses, recognising co‐occurring substance use as a cross‐cutting factor across all formulation domains and explicitly incorporating patient experience and psychological processes are crucial to improving suicide risk assessment and safety planning. An extended 5Ps model, informed by the IMV framework, offers a practical way to enhance risk formulation and support both non‐stigmatising practice and integrated, effective care.

## 9 Relevance for Mental Health Nursing

- Co‐occurring substance use disorders are prevalent among those accessing mental health services.
- People with co‐occurring substance use and mental health conditions represent a high‐risk group for suicide and should be prioritised for proactive, integrated care.
- Mental health nurses can use an extended 5Ps framework that includes Patient experience, identifies a lack or loss of Protective factors, and is informed by the IMV model to conduct more nuanced, person‐centred risk formulations.
- Service development should focus on reducing stigma and equipping nurses to explore psychological processes such as hopelessness and mental pain, as well as the impact of service experiences on suicidal distress.

## Author Contributions

In accordance with IJMHN criteria, the contributions of each author are as follows: **Rebecca Marshall:** conceptualisation of the review; development of research question and aims; design of search strategy; screening, data extraction and quality appraisal; analysis and synthesis of findings; drafting and critical revision of the manuscript; approval of the final version. **Anita Green:** methodological guidance on integrative review design; independent quality appraisal of a subset of studies; screening of a subset of studies for inclusion/exclusion; critical review of clinical and theoretical interpretations; revision of the manuscript; approval of the final version. **Magdalena Zasada:** methodological guidance on integrative review design; screening of a subset of studies for inclusion/exclusion; critical review of clinical and theoretical interpretations; revision of the manuscript; approval of the final version. **Caroline Nicholson:** methodological guidance on integrative review design; screening of a subset of studies for inclusion/exclusion; critical review of clinical and theoretical interpretations; revision of the manuscript; approval of the final version.

## Funding

The authors have nothing to report.

## Conflicts of Interest

The authors declare no conflicts of interest.

## Data Availability

The data that supports the findings of this study are available in Supporting Information of this article.
